# Discovery of (phenylureido)piperidinyl benzamides as prospective inhibitors of bacterial autolysin E from *Staphylococcus aureus*

**DOI:** 10.1080/14756366.2018.1493474

**Published:** 2018-08-24

**Authors:** Jure Borišek, Sara Pintar, Mitja Ogrizek, Simona Golič Grdadolnik, Vesna Hodnik, Dušan Turk, Andrej Perdih, Marjana Novič

**Affiliations:** a National Institute of Chemistry, Ljubljana, Slovenia;; b Department of Biochemistry, Molecular and Structural Biology, Jozef Stefan Institute, Ljubljana, Slovenia;; c Jozef Stefan International Postgraduate School, Ljubljana, Slovenia;; d Biotechnical Faculty, Infrastructural Center for Surface Plasmon Resonance, Ljubljana, Slovenia;; e Centre of Excellence for Integrated Approaches in Chemistry and Biology of Proteins, Ljubljana, Slovenia

**Keywords:** Autolysin E, glycoside hydrolase, virtual screening, SPR, STD NMR

## Abstract

Autolysin E (AtlE) is a cell wall degrading enzyme that catalyzes the hydrolysis of the β-1,4-glycosidic bond between the *N*-acetylglucosamine and *N*-acetylmuramic acid units of the bacterial peptidoglycan. Using our recently determined crystal structure of AtlE from *Staphylococcus aureus* and a combination of pharmacophore modeling, similarity search, and molecular docking, a series of (Phenylureido)piperidinyl benzamides were identified as potential binders and surface plasmon resonance (SPR) and saturation-transfer difference (STD) NMR experiments revealed that discovered compounds bind to AtlE in a lower micromolar range. (phenylureido)piperidinyl benzamides are the first reported non-substrate-like compounds that interact with this enzyme and enable further study of the interaction of small molecules with bacterial AtlE as potential inhibitors of this target.

## Introduction

In the last decade resistance to antibiotics and other antimicrobial compounds became a global problem with increasing importance^1^. One of the promising strategies to confront aforementioned challenge is to investigate new antibiotic targets aiming at discovery of new classes of antibiotics^2^. In particular, remodeling and hydrolysis of cell wall remains largely unexploited territory as opposed to widely explored area of targeting peptidoglycan synthesis pathways^3^. Autolysin E (AtlE) from *Staphylococcus aureus* is one of the peptidoglycan degrading enzymes^4^ and its inhibition could pave the way to potential novel class of antimicrobial agents.

*Staphylococcus aureus* is a widespread gram-positive pathogen of humans and animals. In most cases, it lives as a commensal organism on the skin and mucous membranes and poses no threat to healthy individuals. However, when it enters into the body through breaches in skin or membranes, it may pose a serious risk especially for immunocompromised individuals. It can cope with hostile conditions encountered in the bloodstream of the living host, a scarce supply of certain nutrients, attacks of the immune system and anti-infective measures undertaken in the clinical field. List of infections it causes includes bacteremia, infective endocarditis, impetigo, surgical site infections, cutaneous abscesses, purulent cellulitis, osteomyelitis, septic arthritis, prosthetic device infections, and toxic shock syndrome^5^^,^^6^. Another feature that makes it even more difficult to treat is its ability to form biofilm. Biofilm is a community of microorganism that is attached to the surface and plays a significant role in persistence of bacterial infections^7^. Bacteria within biofilms are several orders of magnitude more resilient to antibiotics, compared with planktonic bacteria^8^. The huge repertoire of different virulence factors and additional supportive gene products that increase its capability to survive within the living host make *S. aureus* one of the most threatening microorganisms causing hospital and community-acquired infections^9^.

Wide-spread use of antibiotics in recent decades has resulted in emergence of antibiotic and multiple antibiotic resistant strains, such as methicillin (MRSA) and vancomycin resistant *S. aureus*. Before 1980s MRSA was considered a healthcare-associated disease, however there has been a dramatic increase in the number of community-based infections due to it^10^. For most of the known antibiotics, strains resistant to each, have been isolated^11^. The threat of the spread of even more resistant *S. aureus* strains urges the development of new antibiotics targeting this organism.

The genome of *S. aureus* strain Mu50 codes for five *N*-acetyl-glucosaminidases, one of them being AtlE, which belongs to the glycoside hydrolases (GH) 73 family. During growth and division bacteria have to synthesise and degrade peptidoglycan. Cells with inactivated *N*-acetyl-glucosaminidases have morphological defects as a consequence of impaired ability to increase in size after division and adopt correct mature shape^12^^,^^13^. Further it has been shown that some autolysins have a role in concealing bacteria from the receptor proteins that detect peptidoglycan and thus enable the pathogen to evade the immune system of the host^14^. Therefore, enzymes involved in the cell wall degradation can be considered as novel, valuable extracellular drug/vaccine targets. It may seem at a first glance contradictory that blocking the enzyme responsible for the degradation of the bacterial cell wall would have antimicrobial effect. However, as described above, the bacteria needs to degrade peptidoglycan in certain stages of growth and division, thus the inhibition of such enzymes would be beneficial. Interestingly, also an alternative molecular engineering approach was developed to enhance the bacteriolytic activity of autolysins and thus make them promising antimicrobial agents^15^.

**Figure 1.**
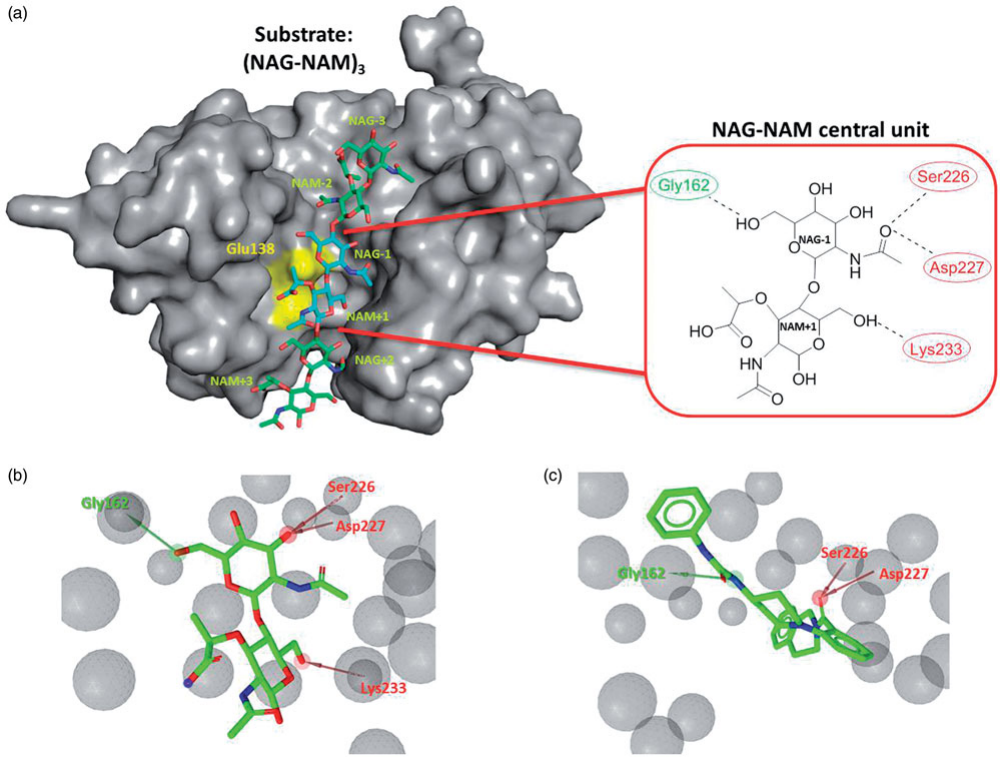
(a) Remodeled substrate in AtlE (PDB: 4PIA) and schematic presentation of the selected NAG-NAM central unit in AtlE for further virtual screening campaign; yellow region represents catalytic amino acid Glu138. Dotted lines represent hydrogen bonds between the central substrate unit and the protein active site residues. Green and red residues represent hydrogen bond acceptors and donors, respectively. (b) LigandScout structure-based pharmacophore for the modeled central NAG-NAM ligand within the AtlE binding site and (c) micromolar hit **3** obtained from the first screening campaign aligned to the pharmacophore model. Green arrows represent hydrogen bond donors, red arrows hydrogen bond acceptors and gray spheres exclusion volume spheres. Pharmacophore model comprised one hydrogen bond donor (interaction with Gly162) and three hydrogen bond acceptors, describing possible interactions with Ser226, Asp227 and Lys233.

AtlE from *S. aureus* is an enzyme from the GH73 family^16^. In our previous studies, the crystal structure of AtlE and its structures with fragments of its substrate have been determined and thereby the binding groove for the substrate has been experimentally identified^4^. AtlE has a binding site that can accommodate three NAG-NAM units of its natural peptidoglycan substrate and cleaves the β-1,4-glycosidic bond between the *N*-acetylglucosamine (NAG) and *N*-acetylmuramic acid (NAM). Furthermore, active site residue Glu138 was confirmed as crucial for the activity of the enzyme (Figure 1)^4^^,^^17^.

In order to discover AtlE inhibitors, several computational structure-based drug design tools were employed. Binding affinities of the most promising virtual hits obtained by iterative processes of pharmacophore modeling, similarity search and molecular docking were determined experimentally using surface plasmon resonance (SPR) and saturation-transfer difference (STD) NMR experiments.

## Materials and methods

### Investigation of the AtlE binding site and pharmacophore-based virtual screening experiments

In order to determine the starting point for the virtual screening, experimentally remodeled AtlE substrate was derived from previous study^4^. As remodeled AtlE substrate contains 3 (NAG-NAM) units, we focused on the central NAG-NAM unit being bound near the catalytic Glu138 residue. The remodeled substrate structure of the NAG-NAM unit in the AtlE binding site (PDB ID: 4PIA) was used to explore interactions using LigandScout software^18^ and to derive structure-based pharmacophore for the NAG-NAM ligand (see Figure 1). The pharmacophore model was used to screen approximately 5 million commercially available compounds, all of which were previously converted into multifunctional format (25 conformers for each compound in the database) using the LigandScout screening module^19^. The conformers of the molecules in the screening library were generated using the idbgen module available in Ligandscout, coupled with the OMEGA software^20^. The default high-throughput settings were used for the library generation: maximum number of output conformers per molecule = 25; RMS threshold to duplicate conformers = 0.8 A; maximum number of all generated conformers per molecule = 30,000; and maximum number of intermediate conformers per molecule = 4000. Pharmacophore Fit scoring function was used to score the matching of the hit molecules to the pharmacophore model. The LigandScout screening procedure retrieved approximately 10,000 hit compounds that were subsequently docked in the AtlE binding site.

### Molecular docking calculations

Molecular docking studies were performed using GOLD Suite v5.1 docking package^21^. Binding site was defined around remodeled substrate coordinates of the NAG-NAM ligand in radius of 12 Å. Validation of the docking settings^22^ was performed by redocking of the NAG-NAM ligand of the remodeled substrate into the AtlE binding site. The observed RMSD of obtained docked poses versus the original NAG-NAM position were within the accepted limits and CHEMPLP scoring function gave the best results.

Next, each ligand was docked into the binding site by the scoring function CHEMPLP and applying the following parameters of the GOLD genetic algorithm: population size = 100, selection pressure = 1.1, no. of Operations = 100,000, no. of islands = 5, Niche Size = 2, migrate = 10, mutate = 95, crossover = 95.42.

### Surface plasmon resonance (SPR) experiments

SPR experiments were performed using a Biacore T100 (GE Healthcare) equipped with a Series S sensor chip CM5 (GE Healthcare). PBS buffer (67 mM Na2HPO4, 12.5 mM KH2PO4, 70 mM NaCl, 0,05% P20, pH 7.4) was used for immobilisation of the protein. AtlE was produced as previously described^1^ and attached to the surface of the chip by amine coupling. The surface of flow cells 1 and 2 was initially activated with a 10-min pulse of a 1:1 mixture of 0.4 M 1-ethyl-3–(3-dimethylaminopropyl)-carbodiimide hydrochloride and 0.1 M N-hydroxysuccinimide, according to manufacturer recommendations. AtlE was diluted into 10 mM sodium acetate buffer, pH 5.8, to a final concentration of 100 µg/mL, and injected twice for 180 s over the second flow cell. Both flow cells were blocked with a 7-min pulse of 1.0 M ethanolamine. The final immobilisation level was about 4100 RU. Screening was run at 25 °C using PBS (67 mM Na2HPO4, 12.5 mM KH2PO4 pH 7.4, 70 mM NaCl, 0.05% P20) supplemented with 1% or 5% DMSO (Merck) as the running buffer. Selected compounds from the virtual screening studies were tested at two different concentrations: 40 and 400 µM. Each compound was injected for 1 min at a flow-rate of 30 µL/min, and the dissociation was monitored for 30 s. Regeneration was not needed, although 30 s of buffer-flow was used to stabilise the surface after each injection. We compared the responses obtained by the injections of the buffer and the two different sample concentrations, and continued the study with the compounds that showed binding. Selected compounds were tested at eight different concentrations (depending on their solubility) in three parallel titrations. KD values were determined by fitting of the data to a 1:1 steady state binding model.

### Nuclear magnetic resonance (NMR) experiments

The high-resolution NMR spectra were recorded on a Varian DirectDrive 800 MHz spectrometer at 25 °C. All data were collected using pulse sequences and phase-cycling routines provided in Varian libraries of pulse programs. The cryogenic triple-resonance NMR probe was used. NMR samples were prepared in a buffer containing 20 mM Tris-D11, 10% glycerol-D8, and 100 mM NaCl in D_2_O, pD 7.2. DSS (0.1 mM) was used as an internal standard. All the spectra were recorded at a protein/ligand ratio of 1:100, the protein concentration was 1 µM and the ligand concentration was 0.1 mM. The STD experiments^23^ were performed with a 8802.8 Hz spectral width, 16384 data points, a saturation time of 2 s, a relaxation delay of 1.3 s, and 3800 scans. Selective saturation was achieved by a train of 50 ms long Gauss-shaped pulses separated by 1 ms delay. Water was suppressed via excitation sculpting. The on-resonance selective saturation of AtlE was applied at –0.9 ppm, which is well separated from the methyl group of compounds at 1.1 ppm. The off-resonance irradiation was applied at 30 ppm for the reference spectrum. Subtraction of the on and off-resonance spectra was performed internally via phase cycling. Spectra were zero-filled twice and apodised by an exponential line-broadening function of 1 Hz.

## Results and discussion

### Design phase I: virtual screening

To identify AtlE inhibitors, our design starting point were the available structural information of the enzyme AtlE (PDB ID: 4PIA) and its remodeled substrate from our previous study^4^. Briefly, the substrate model in the active site cleft of AtlE was constructed by merging information from substrate fragments of superimposed complexes of AtlE and G-type lysozyme (PDB IDs: 4PI7^4^, 4PI9^4^, 3GXR^24^ and 148L^25^). The AtlE substrate model contained 3 NAG-NAM units. The central unit NAG-1–NAM +1 representing the cleavage site was positioned next to the catalytic Glu138^4^. This disaccharide unit was selected to derive structure based pharmacophore for the initiation of the virtual screening campaign (Figure 1(a)) and remodeled^4^ using ligands NAG and MUB from the complexes 3GXR and 148 L, respectively (Figure 1(b)). It contained the interaction pattern with the AtlE based on hydrogen bonds with Gly162, Ser226, Asp227 and Lys233.

To identify molecules that retained molecular recognition pattern of the selected NAG-NAM unit, several *in silico* techniques were employed as powerful drug design tools. First, a structure-based pharmacophore model mimicking the interactions of the selected central NAG-NAM unit with the AtlE binding site was generated using LigandScout software^18^. Pharmacophores consisted of the following features: hydrogen bond acceptor (describing the interaction with the Gly162) and three hydrogen bond donors (observed with Ser226, Ser227 and Lys233) (Figure 1). Exclusion volume spheres were also derived, mimicking the sterical boundary conditions of the AtlE-binding site. Interaction between scissile glycosidic substrate bond and Glu138 was not idetified by LigandScout as potential H-bond pharmacophore feature due to not optimal geometry. This is in accordance with our previous MD simulations of the AtlE bound with (NAG-NAM)_3_ substrate which have suggested a complex role of Glu138 residue in molecular recognition and catalysis^17^.

In order to broaden the chemical space of the resulted virtual hits, we introduced an additional criterion that three out of initial four pharmacophore features had to be satisfied for a compound to be considered as a hit. Subsequently, this pharmacophore model was used in a large scale virtual screening campaign, using available library of approximately 5 million commercially available compounds^19^. This procedure resulted in approximately 10,000 virtual hits from different structural classes that were able to comply with the requested pharmacophore features.

Hits obtained were subsequently docked into the AtlE-binding site using the GOLD molecular docking tool (see Experimental section for GOLD parameter settings) to explore the proposed orientations of these molecules. A successful validation of the docking model was made by redocking of the NAG-NAM molecule in the substrate binding grove. The investigated binding site was defined as a 12 Å spherical cavity around ligand NAG-NAM coordinates. The docking procedure was performed by applying the ChemPLP scoring function^26^, and top 200 ranked docking solutions by scoring function were visually inspected for the fitness and orientation in the AtlE binding site. We were aware that scoring fitness function approach beared certain inherent limitations such as accurate ranking of docking solutions for an investigated ligand and adequate description of entropic contributions^27^. Since no ligands are known for this target, we decided to utilise this option as selection criteria. There are cases in the literature that support the potential of this approach^28^. Finally, 41 compounds from different structural classes were selected from the pool of approximately 200 hits by applying previously derived criteria of fitness, orientation and scoring function, and their binding affinities to AtlE was determined using SPR (see Supplementary Table 1). In the first design phase, several compounds from the diverse structural classes exhibited binding affinity in milimolar range (*K*_d_ > 1000 µM) Table 1.

**Table 1.** Tested compounds with binding affinities to AtlE.

Compound	Structure	*K*_d_ (µM)	Compound	Structure	*K*_d_ (µM)
**1**		>1000	**6**		>1000
**2**		>1000	**7**		>1000
**3**		177	**8**		>1000
**4**		>1000	**9**		>1000
**5**		>1000			

Most promising hit **3** marked in gray.

We were especially pleased to observe that compound **3** from the structural class of (phenylureido)piperidinyl benzamides was found to exhibit promising binding affinity to AtlE in the micromolar range (see Figure 1(c) and Figure S1c for SPR sensorgram of **3**) making it to the best of our knowledge the first identified reported compound that binds this target.

To gain an insight into the molecular recognition of **3** by AtlE molecular docking was applied. A binding mode suggested hydrogen bonds with AtlE residues Gly162, Tyr224, Ser226 and Asp227, hydrophobic interactions with Phe63 and Tyr201, and electrostatic cation-Pi interaction with Lys233 (Figure S1).

### Design phase II: screening and evaluation of a (phenylureido)piperidinyl benzamides focused library

The first phase of our VS campaign, described in Design Phase I, yielded a promising micromolar AtlE ligand, to the best of our knowledge the first ever described small molecule binder for this target. In order to provide initial SAR a focused library of all commercially available, (phenylureido)piperidinyl benzamides was extracted from the eMolecules library.^29^ It consisted of 16 molecules (**10–25**), which were tested for their binding affinity to AtlE by SPR. None of these molecules were present in our initial library of screened compounds. The results of the SPR binding affinity assay are presented in Table 2; SPR sensorgrams for active compounds are shown in Figure S2.

**Table 2.** Compounds from the structural class of (phenylureido)piperidinyl benzamides tested for AtlE binding affinity in SPR experiments.
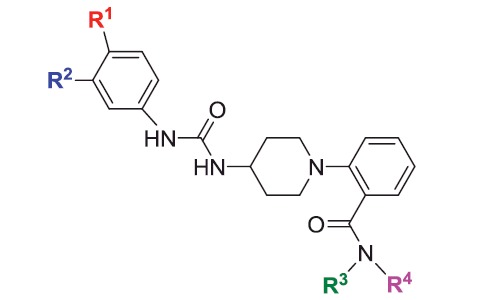

Compd	R^1^	R^2^	R^3^	R^4^	*K*_d_ (µM)
**10**	-H	-Cl			1.9
**11**	-Me	-H		-H	2.4
**12**	-H	-Cl		-H	2.5
**13**	-Me	-H		-H	6.1
**14**	-Me	-H		-H	11.3
**15**	-Me	-H		-H	11.7
**16**	-Me	-H			18.2
**17**	-H	-H		-H	21.1
**18**	-Me	-H		-Et	38.8
**3**	-H	-H			177
**19**	-Me	-H		-H	>1000
**20**	-Me	-H		-Me	>1000
**21**	-H	-H			>1000
**22**	-H	-H		-Et	>1000
**23**	-Me	-H		-H	>1000
**24**	-H	-H		-H	>1000
**25**	-Me	-H			>1000

Additionally, SPR experiments revealed fast and tight binding to the AtlE at low micromolar concentrations. All commercially available compounds possessing AtlE binding affinity were characterised for their identity by HR-MS techniques (see Supplementary Information).

Our further SPR measurements of the focused library yielded additional nine compounds with the binding affinity to AtlE in the low micromolar range from the same structural class of (phenylureido)piperidinyl benzamides (Table 2). This enabled rough estimation of SAR properties required for binding to this target.

**Figure 2.**
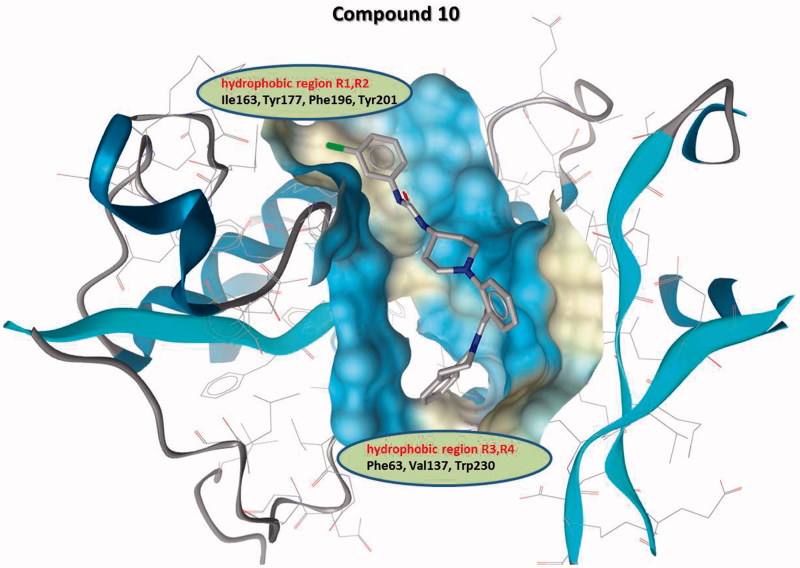
Hydrophilic and hydrophobic regions of AtlE binding surface for modeled compound **10** are depicted in blue and yellow, respectively. Hydrophobic region R1, R2 and R3, R4 represent the area where substituents of (phenylureido)piperidinyl benzamides R1 and R2, and R3, R4 bind, respectively.

**Figure 3.**
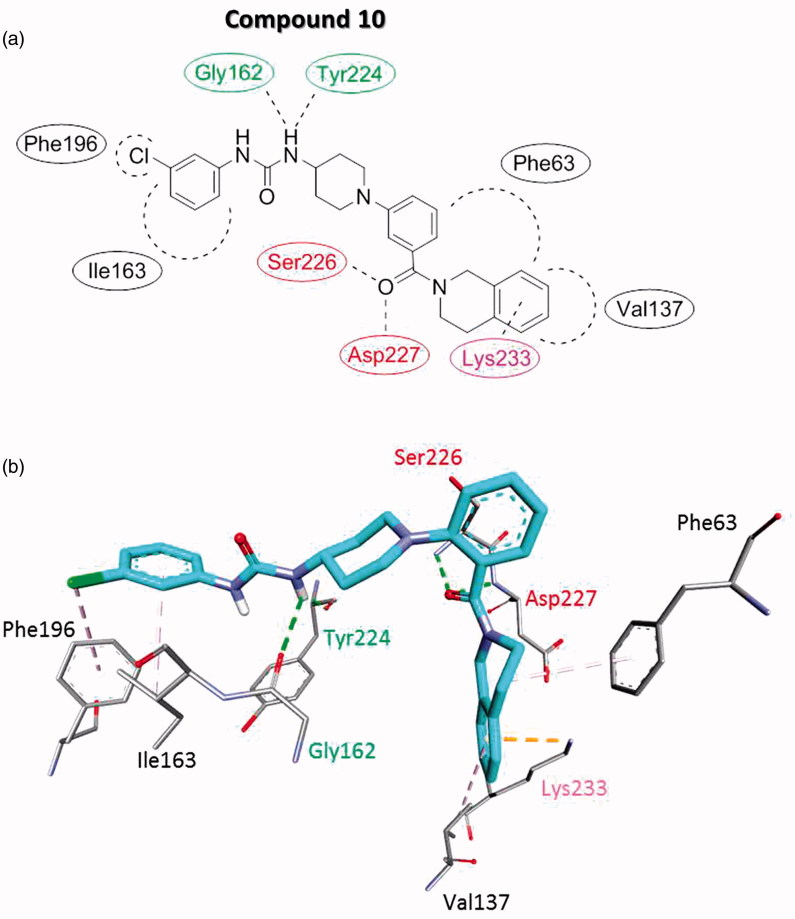
Proposed interaction binding pattern between the active (phenylureido)piperidinyl benzamides compound **10** and the AtlE binding site based on molecular docking calculations, depicted in (a) 2D and (b) 3D. It comprises two hydrogen bonds acceptors (Gly162 and Tyr224), two hydrogen bond donors (Ser226 and Asp227), several hydrophobic interactions with Phe63, Val137, Ile163, and Phe196 and Lys233 electrostatic cation-Pi interaction. Green residue represents hydrogen bond acceptor, red residues hydrogen bond donors, black residues form hydrophobic interactions and magenta residue forms electrostatic cation–Pi interaction.

Based on the binding affinities and on the molecular docking calculations of compounds **3**, **10** and **16**, we concluded that the chlorine substituent at the R2 position is the most important for binding affinity as the R1 methyl containing **16** has 10 times lower affinity (*K*_d_ = 18.2 µM) and R1, R2 non-substituted **3** (*K*_d_ = 177 µM) has 100 times lower affinity than **10** (*K*_d_ = 1.9 µM), respectively. We could hypothesise that R1 and R2 substituents bind into the hydrophobic region of AtlE-binding site comprising Ile163, Tyr177, Phe196 and Tyr201 residues (Figures 2 and 3).

Compounds with cyclic lipophilic/aromatic fragments on the R3 and/or the R4 display a higher affinity (*K*_d_ = low µM range) than compounds **22**, **23**, **24** (*K*_d_ > 1000 µM) with alkyl fragments on the R3 and/or R4 positions. The models suggested that the substituents on the R3 and/or R4 positions bind into the elongated hydrophobic region, comprisedby Phe63, Val137, and Trp230 residues and also might form additional electrostatic interactions with Glu138 and Lys233. Most of the active (phenylureido)piperidinyl benzamides compounds form hydrogen bonds with Gly162, Gly164, Tyr224, Ser226 and Asp227.

Binding model of the ligands in AtlE binding site based on the molecular docking calculations was similar for all the (phenylureido)piperidinyl benzamides compounds. It is schematically presented for the model compound **10** (Figure 3).

The AtlE substrate binding site is quite extensive as it can accommodate the substrate with six aminosugar NAG-NAM units. Thus alternative binding modes seemed also possible for the relatively small molecule as **10**^30^. Nevertheless, the interaction binding pattern of compound **10** was well defined. It comprised two hydrogen bonds acceptors (Gly162 and Tyr224), two hydrogen bond donors (Ser226 and Asp227), and several hydrophobic interactions with Phe63, Val137, Ile163, and Phe196 and Lys233 electrostatic cation–Pi interaction. Compared to the initial structure-based pharmacophore for the NAG-NAM (see Figure 1), **10** retained the binding pattern of one hydrogen bond donor (Gly162) and two hydrogen bond acceptors (Ser226 and Asp227).

We unsuccessfully attempted to perform enzymatic assays due to poor compounds solubility. In order for this assays to be successful the concentration of the inhibitor needs to be above its solubility in used assay settings. However, (phenylureido)piperidinyl benzamide are the first known series of nonsubstrate-like small molecules with confirmed binding to AtlE and are as such a good starting point for subsequent optimisation studies. To confirm the SPR results that revealed binding affinity of selected compounds in micro-molar concentration range, we have applied the STD NMR method, which provides well recognised alternative binding experiments^31^.

### STD NMR binding measurements

Additionally, we tested the binding nature of selected binders with STD NMR spectroscopy. For the STD NMR experiments, we selected the most soluble compounds among the active ones, i.e. two (phenylureido)piperidinyl benzamides, **13** and **14** (Table 2). Due to overall low solubility of the compounds from this class their binding to AtlE was probed at experimental conditions for qualitative STD detection along with long selective saturation time of AtlE. We obtained weak STD signals in the aromatic region of compounds (Figure S3), which indicates that the compounds **13** and **14** bind to AtlE.

In addition to the poor solubility of the compounds (up to 100 µM), their low binding affinities (*K*_d_ = 10 µM) near the limit of detection of the method^23^ may provide explanation for weak STD signals. Furthermore, due to the presence of methyl groups in ligands, we could not irradiate signals of protein in positive ppm range to improve transfer of saturation along protein.

## Conclusions

In conclusion, using recently determined crystal structures of AtlE from *S. aureus* coupled with pharmacophores and molecular docking, a small series of (phenylureido)piperidinyl benzamides was discovered. Virtual screening and subsequent SPR and STD NMR binding experiments revealed that compounds interact with the AtlE in the lower micromolar range. (phenylureido)piperidinyl benzamides thus represent the first reported non-substrate like compounds that interact with this bacterial target with shown direct binding data obtained by two independent biophysical experiments. Thus, these compounds offer promise to pave the way to the first hit compounds of this cell wall degrading enzyme and enable its validation as drug target.

## Supplementary Material

Supplemental Material
