# Supplementary material for: Discovery of (phenylureido)piperidinyl benzamides as prospective inhibitors of bacterial autolysin E from Staphylococcus aureus
Source: J Enzyme Inhib Med Chem. 2018 Aug 24;33(1):1239–47. doi: 10.1080/14756366.2018.1493474 (PMC6116672; doi:10.1080/14756366.2018.1493474)
Supplement: Supplemental Material [file IENZ_A_1493474_SM1691.zip › IENZ_1493474_Supplementary Material/IENZ_1505794_Supplementary Material.pdf]

Supporting information

Discovery of (phenylureido)piperidinyl Benzamides as Prospective Inhibitors of Bacterial Autolysin E from *Staphylococcus aureus*

Jure Borišek[†], Sara Pintar[‡|], Mitja Ogrizek[†], Simona Golič Grdadolnik[†], Vesna Hodnik[⊥], Dušan Turk[‡,§], Andrej Perdih[†] and Marjana Novič[†*]

[†]*National Institute of Chemistry, Hajdrihova 19, SI-1001 Ljubljana, Slovenia*
[‡]*Department of Biochemistry, Molecular and Structural Biology, Jozef Stefan Institute, Jamova cesta 39, SI-1000 Ljubljana, Slovenia*
[|]Jozef Stefan International Postgraduate School, Jamova cesta 39, SI-1000 Ljubljana, Slovenia
[⊥]*Biotechnical Faculty, Infrastructural Center for Surface Plasmon resonance, 1000 Ljubljana, Slovenia*
[§]*Centre of Excellence for Integrated Approaches in Chemistry and Biology of Proteins, Jamova cesta 39, SI-1000 Ljubljana, Slovenia*

Corresponding Author

*E-mail: marjana.novic@ki.si

Table of Contents

Proposed Binding Mode of the Hit Compound 3 .... S3

SPR Sensorgrams S4

STD NMR and ^{1}H NMR Specters for Selected Compounds S5

List of Tested Compounds S6

Elemental Composition Reports S13

Compound 3 .... S13

Compound 10 .... S14

Compound 11 .... S15

Compound 12 .... S16

Compound 13 .... S17

Compound 14 .... S18

Compound 15 .... S19

Compound 16 .... S20

Compound 17 .... S21

Compound 18 .... S22

Proposed Binding Mode of the Hit Compound 3

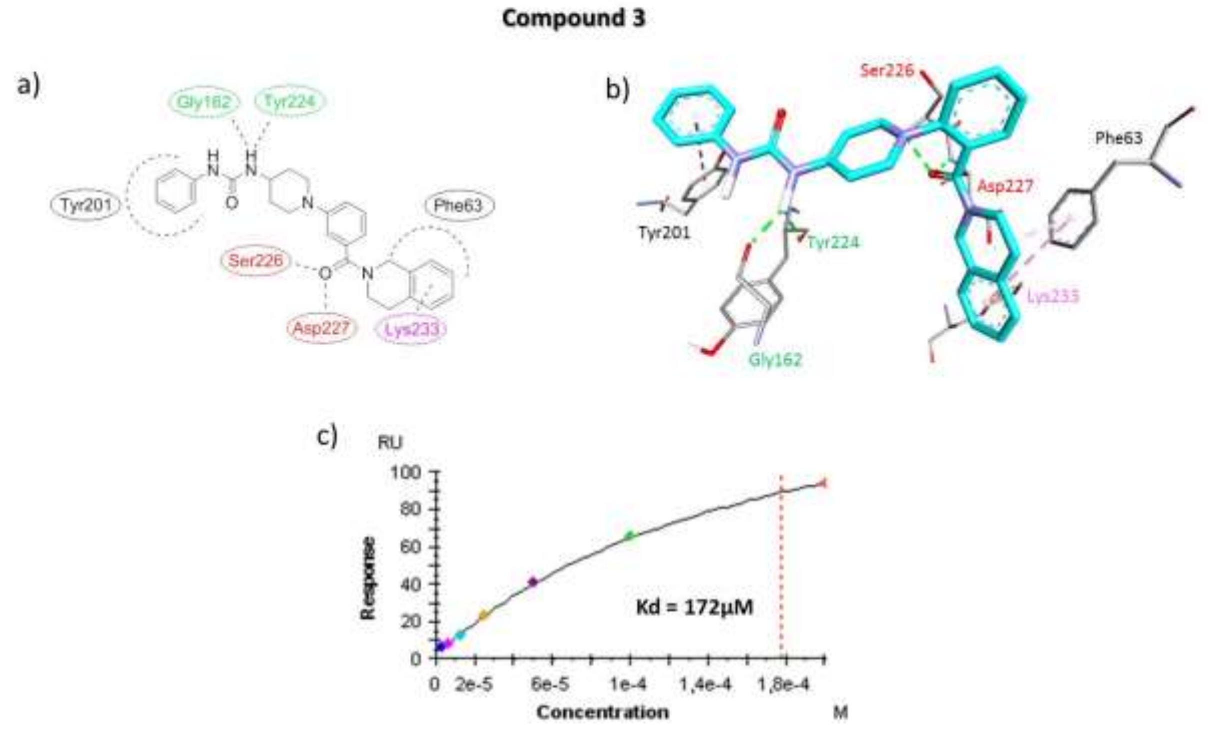

Figure S1. Proposed binding mode of the hit compound **3** in the AtlE binding site with interacting residues depicted in a) 2D, b) 3D and c) obtained SPR sensorgram. Gly162, Tyr224, Ser226 and Asp227 form hydrogen bonds, Phe63 and Tyr201 hydrophobic interactions, and Lys233 electrostatic cation-Pi interaction. Green residue represents hydrogen bond acceptor, red residues hydrogen bond donors, black residues form hydrophobic interactions and magenta residue forms electrostatic cation-Pi interaction.

SPR Sensorgrams

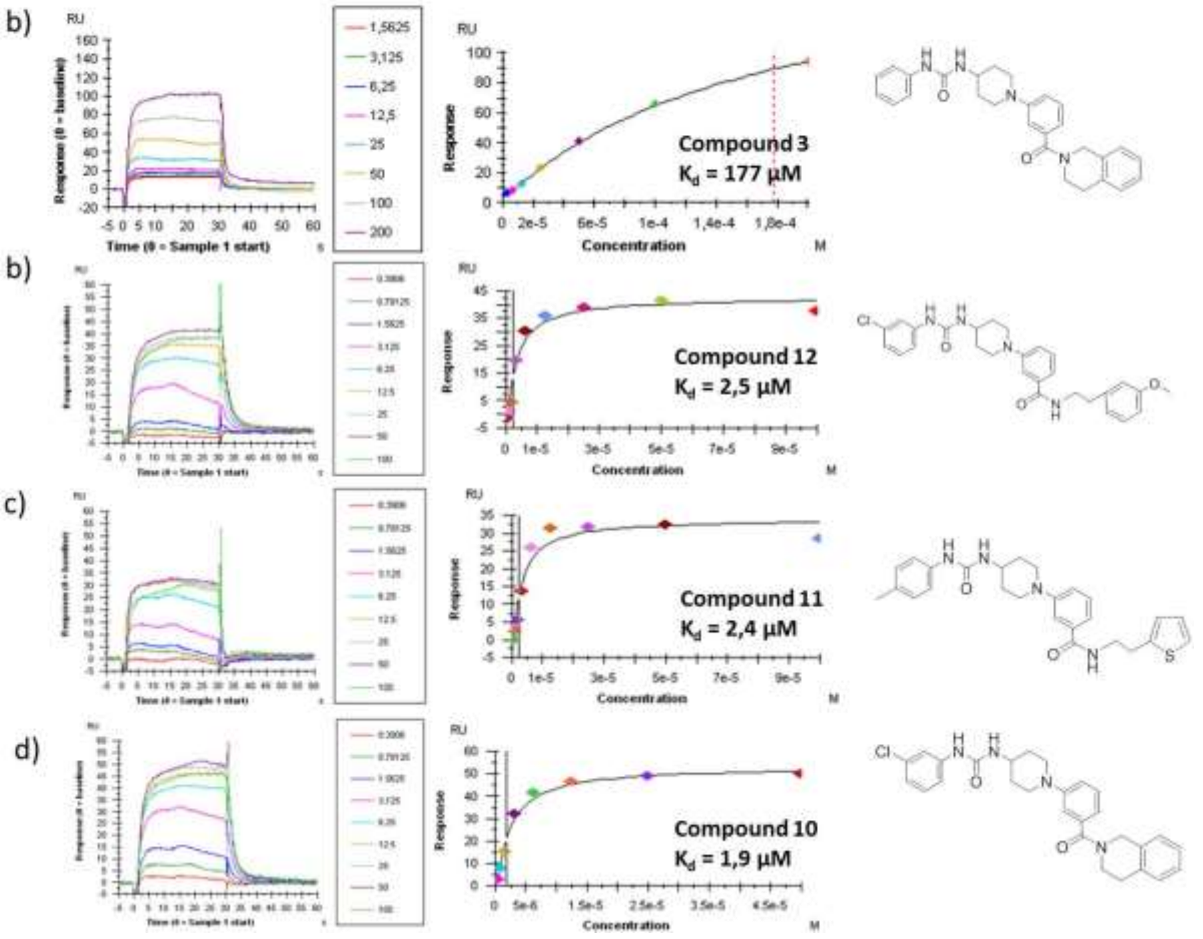

Figure S2. SPR sensorgrams for (phenylureido)piperidinyl benzamide compounds **3**, **10**, **11** and **12** of the focused library with corresponded fitted K_d values.

STD NMR and ^{1}H NMR Specters for Selected Compounds

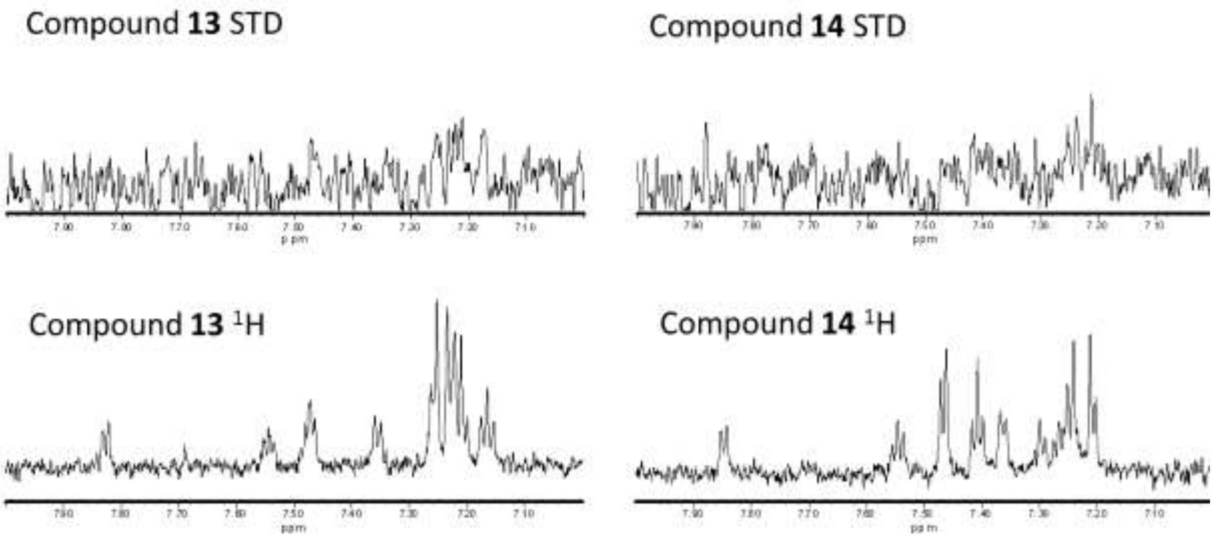

Figure S3. STD NMR and ^{1}H NMR specters for selected most soluble compounds **13** and **14**. Around 7.2 ppm in aromatic region STD effect could be observed.

List of Tested Compounds

Table S1. 41 selected compounds for the binding affinity testing with corresponding SMILES codes.

1	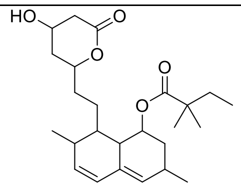<br>CCC(C)(C(OC1CC(C=C2C=CC(C(C12)CCC3CC(CC(O3)=O)O)C)C)=O)C<br>Vendor: Vitas M Labs
2	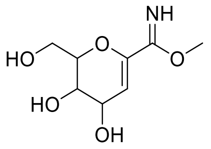<br>COC(C1=CC(O)C(O)C(CO)O1)=N<br>Vendor: ChemDiv
3	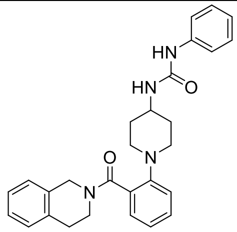<br>O=C(NC1=CC=CC=C1)NC2CCN(C3=CC=CC=C3C(N4CCC5=C(C=CC=C5)C4)=O)CC2<br>Vendor: ChemDiv
4	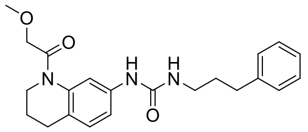<br>COCC(N1CCCC2=C1C=C(C=C2)NC(NCCCC3=CC=CC=C3)=O)=O<br>Vendor: Life Chemicals
5	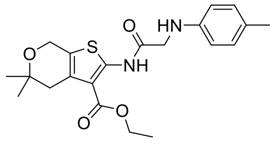<br>CCOC(C1=C(SC2=C1CC(C)(OC2)C)NC(CNC3=CC=C(C=C3)C)=O)=O<br>Vendor: Vitas M Labs

6	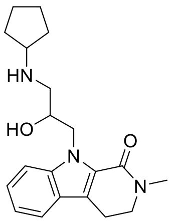<br>CN1CCC2=C(C1=O)N(C3=C2C=CC=C3)CC(CNC4CCCC4)O<br>Vendor: InterBioScreen
7	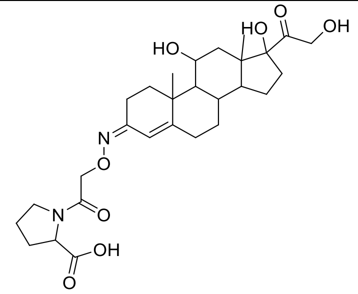<br>CC12CC(C3C(C1CCC2(C(CO)=O)O)CCC4=C/C(CCC34C)=N\OCC(N5CCCC5C(O)=O)=O)O<br>Vendor: InterBioScreen
8	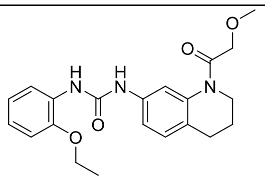<br>CCOC1=C(C=CC=C1)NC(NC2=CC=C3CCCN(C3=C2)C(COC)=O)=O<br>Vendor: Life Chemicals
9	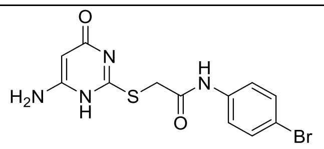<br>NC1=CC(N=C(SCC(NC2=CC=C(C=C2)Br)=O)N1)=O<br>Vendor: ChemDiv
10	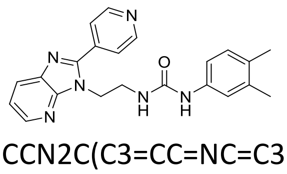<br>CC1=CC=C(C=C1C)NC(NCCN2C(C3=CC=NC=C3)=NC4=CC=CN=C24)=O<br><br>Vendor: ChemDiv
11	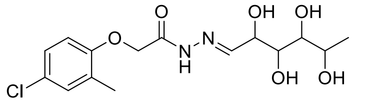<br>CC(C(C(C(/C=N/NC(COC1=CC=C(C=C1C)Cl)=O)O)O)O)O<br>Vendor: ChemDiv
12	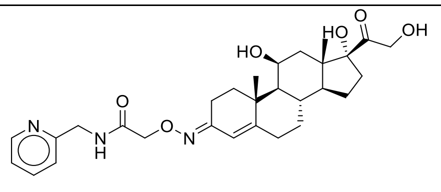

	C[C@]12C[C@@H]([C@H]3[C@H]([C@@H]1CC[C@@]2(C(CO)=O)O)CCC4=C/C(CC[C@]34C)=N/O<br>CC(NCc5ncccc5)=O)O<br>Vendor: Molport
13	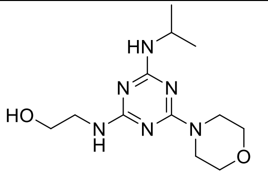<br>CC(NC1=NC(NCCO)=NC(N2CCOCC2)=N1)C<br><br>Vendor: ChemDiv
14	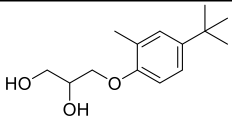<br>CC1=CC(C(C)(C)C)=CC=C1OCC(CO)O<br>Vendor: Enamine
15	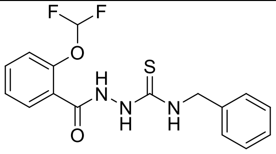<br>FC(OC1=CC=CC=C1C(NNC(NCC2=CC=CC=C2)=S)=O)F<br>Vendor: Vitas-M
16	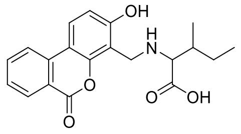<br>CCC(C(C(O)=O)NCC1=C2OC(C3=C(C2=CC=C1O)C=CC=C3)=O)C<br><br>Vendor: ChemDiv
17	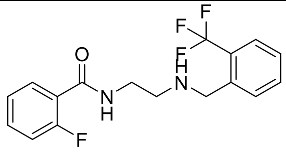<br>FC1=CC=CC=C1C(NCCNCC2=C(C(F)(F)F)C=CC=C2)=O<br><br>Vendor: Vitas-M
18	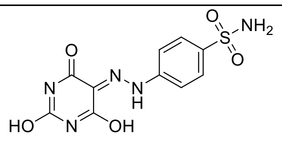<br>NS(=O)(C1=CC=C(C=C1)N/N=C2C(O)=NC(O)=NC\2=O)=O<br>Vendor: ChemDiv
19	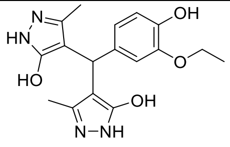<br>CCOC1=C(C=CC(C(C2=C(NN=C2C)O)C3=C(NN=C3C)O)=C1)O<br>Vendor: ChemDiv

20	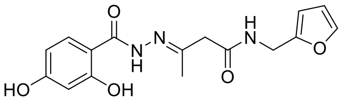<br>C/C(CC(NCC1=CC=CO1)=O)=N\NC(C2=CC=C(C=C2O)O)=O<br>Vendor: ChemDiv
21	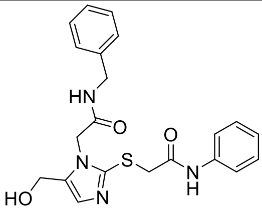<br>OCC1=CN=C(N1CC(NCC2=CC=CC=C2)=O)SCC(NC3=CC=CC=C3)=O<br><br>Vendor: ChemDiv
22	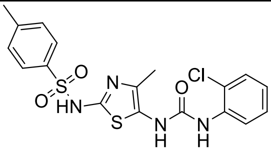<br>CC1=C(SC(NS(=O)(C2=CC=C(C=C2)C)=O)=N1)NC(NC3=CC=CC=C3Cl)=O<br><br>Vendor: Vitas-M
23	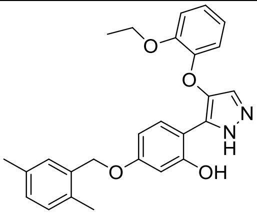<br>CCOC1=CC=CC=C1OC2=C(C3=CC=C(C=C3O)OCC4=CC(C)=CC=C4C)NN=C2<br>Vendor: Vitas-M
24	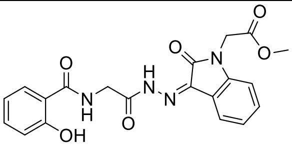<br>COC(CN1C(/C(C2=C1C=CC=C2)=N\NC(CNC(C3=CC=CC=C3O)=O)=O)=O)=O<br>Vendor: Vitas-M
25	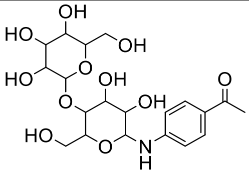<br>CC(C1=CC=C(C=C1)NC2OC(C(C(C2O)O)OC3OC(C(C(C3O)O)O)CO)CO)=O<br><br>Vendor: Vitas-M
26	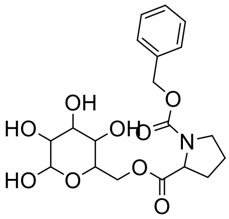

	OC1OC(C(C(C1O)O)O)COC(C2CCCN2C(OCC3=CC=CC=C3)=O)=O<br>Vendor: InterBioScreen
27	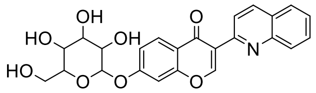<br>OCC1OC(C(C(C1O)O)O)OC2=CC=C3C(OC=C(C4=NC5=CC=CC=C5C=C4)C3=O)=C2<br>Vendor: InterBioScreen
28	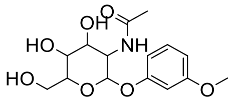<br>COC1=CC=CC(OC2OC(C(C(C2NC(C)=O)O)O)CO)=C1<br>Vendor: Vitas-M
29	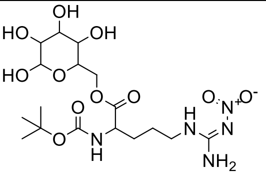<br>CC(C)(OC(NC(C(OCC1OC(C(C(C1O)O)O)O)=O)CCCN/C(N)=N\[N+]([O-])=O)=O)C<br>Vendor: InterBioScreen
30	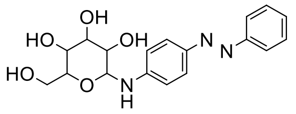<br>OCC1OC(C(C(C1O)O)O)NC2=CC=C(/N=N/C3=CC=CC=C3)C=C2<br>Vendor: ChemDiv
31	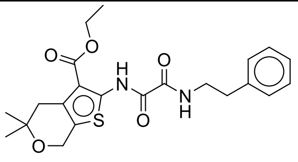<br>CCOC(c1c2c(COC(C)(C2)C)sc1NC(C(NCCc3ccccc3)=O)=O)=O<br>Vendor: Vitas-M
32	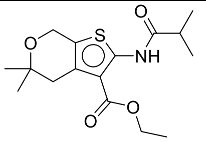<br>CCOC(c1c2c(COC(C)(C2)C)sc1NC(C(C)C)=O)=O<br>Vendor: ChemDiv
33	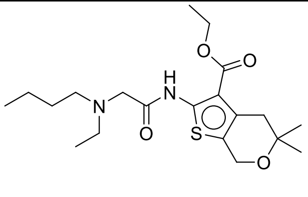<br>CCCCN(CC(Nc1c(C(OCC)=O)c2c(COC(C)(C2)C)s1)=O)CC<br>Vendor: Vitas-M

34	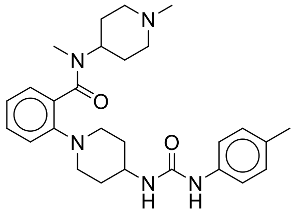<br>CN(C(c1c(N2CCC(NC(Nc3ccc(C)cc3)=O)CC2)cccc1)=O)C4CCN(CC4)C<br>Vendor: ChemDiv
35	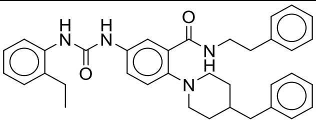<br>CCc1c(NC(Nc2cc(C(NCCc3ccccc3)=O)c(N4CCC(CC4)Cc5ccccc5)cc2)=O)cccc1<br>Vendor: ChemDiv
36	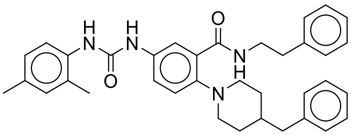<br>Cc1cc(C)c(NC(Nc2cc(C(NCCc3ccccc3)=O)c(N4CCC(CC4)Cc5ccccc5)cc2)=O)cc1<br>Vendor: ChemDiv
37	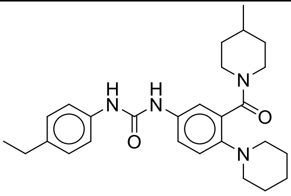<br>CCc1ccc(NC(Nc2cc(C(N3CCC(CC3)C)=O)c(N4CCCCC4)cc2)=O)cc1<br>Vendor: ChemDiv
38	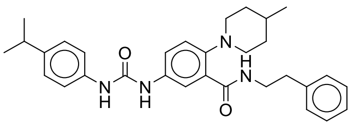<br>CC(c1ccc(NC(Nc2cc(C(NCCc3ccccc3)=O)c(N4CCC(CC4)C)cc2)=O)cc1)C<br>Vendor: ChemDiv
39	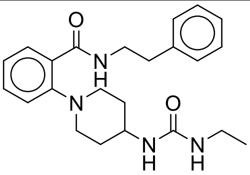<br>CCNC(NC1CCN(c2c(C(NCCc3ccccc3)=O)cccc2)CC1)=O<br>Vendor: ChemDiv
40	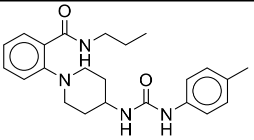

	CCCNC(c1c(N2CCC(NC(Nc3ccc(C)cc3)=O)CC2)cccc1)=O<br>Vendor: ChemDiv
41	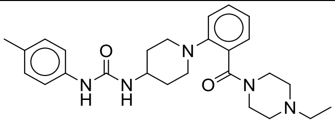<br>CCN1CCN(C(c2c(N3CCC(NC(Nc4ccc(C)cc4)=O)CC3)cccc2)=O)CC1<br>Vendor: ChemDiv

Elemental Composition Reports

Compound 3

Elemental Composition Report — Page 1

Single Mass Analysis
Tolerance = 10.0 PPM / DBE: min = -1.5, max = 50.0
Element prediction: Off
Number of isotope peaks used for i-FIT = 3

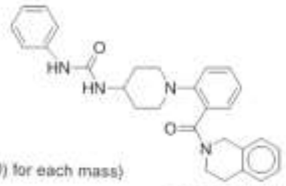

mass = 454.24

Monoisotopic Mass, Even Electron Ions
1485 formula(e) evaluated with 13 results within limits (all results (up to 1000) for each mass)
Elements Used:
C: 0-100 H: 0-120 N: 0-20 O: 0-20
V012-5741 15 (0.609) Cm (15:17-35:42)
1: TOF MS ES+

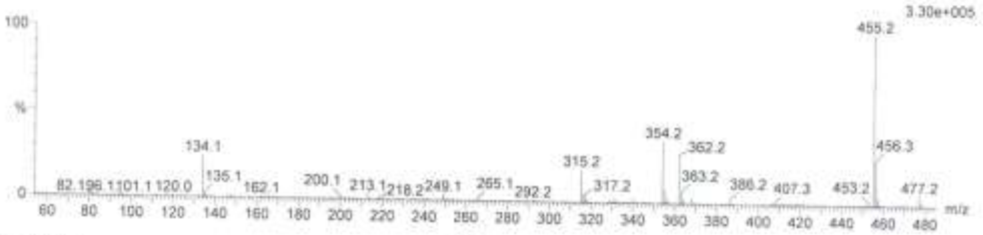

Mass	Calc. Mass	mDa	PPM	DBE	i-FIT	i-FIT (Norm)	Formula
Minimum:				-1.5			
Maximum:		5.0	10.0	50.0			
455.2448	455.2447	0.1	0.2	15.5	308.8	4.6	C28 H31 N4 O2 / MH+
	455.2452	-0.4	-0.9	8.5	310.7	6.5	C13 H27 N16 O3
	455.2439	0.9	2.0	3.5	311.4	7.1	C12 H31 N12 O7
	455.2434	1.4	3.1	10.5	307.6	3.4	C27 H35 O6
	455.2466	-1.8	-4.0	2.5	308.8	4.6	C16 H35 N6 O9
	455.2425	2.3	5.1	-1.5	312.1	7.9	C11 H35 N8 O11
	455.2420	2.8	6.2	16.5	307.8	3.5	C24 H27 N10
	455.2479	-3.1	-6.8	7.5	307.3	3.1	C17 H31 N10 O5
	455.2412	3.6	7.9	4.5	313.5	9.2	C8 H27 N18 O5
	455.2487	-3.9	-8.6	19.5	310.2	5.9	C33 H31 N2
	455.2407	4.1	9.0	11.5	304.8	0.5	C23 H31 N6 O4
	455.2492	-4.4	-9.7	1.5	305.9	1.7	C20 H39 O11
	455.2492	-4.4	-9.7	12.5	306.6	2.4	C18 H27 N14 O

Compound 10

Elemental Composition Report

Page 1

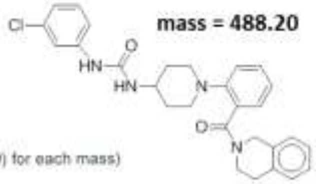

mass = 488.20

Single Mass Analysis
Tolerance = 10.0 PPM / DBE: min = -1.5, max = 50.0
Element prediction: Off
Number of isotope peaks used for i-FIT = 3

Monoisotopic Mass, Even Electron Ions
1419 formula(e) evaluated with 13 results within limits (all results (up to 1000) for each mass)
Elements Used:
C: 0-100 H: 0-120 N: 0-20 O: 0-20 Cl: 1-1
V015-5724 31 (1.274) Cm (31:39-8:23)
1: TOF MS ES+

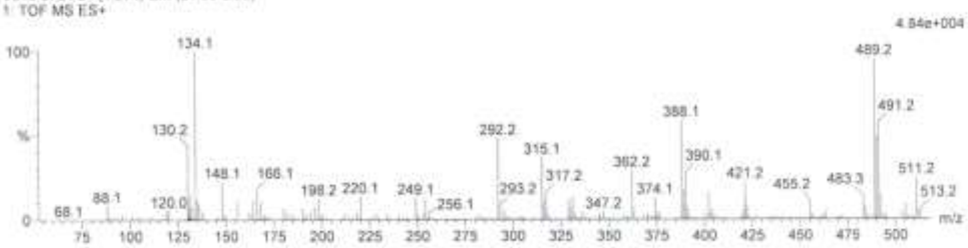

Minimum:				-1.5			
Maximum:		5.0	10.0	50.0			
Mass	Calc. Mass	mDa	PPM	DBE	i-FIT	i-FIT (Norm)	Formula
489.2058	489.2098	-4.0	-8.2	19.5	177.7	1.2	C33 H30 N2 Cl
	489.2057	0.1	0.2	15.5	178.3	1.8	C28 H30 N4 O2 Cl
	489.2044	1.4	2.9	10.5	178.5	2.0	C27 H34 O6 Cl
	489.2030	2.8	5.7	16.5	178.8	2.3	C24 H26 N10 Cl
	489.2017	4.1	8.4	11.5	179.0	2.5	C23 H30 N6 O4 Cl
	489.2103	-4.5	-9.2	12.5	179.7	3.2	C18 H26 N14 O Cl
	489.2103	-4.5	-9.2	1.5	179.7	3.2	C20 H38 O11 Cl
	489.2089	-3.1	-6.3	7.5	179.9	3.4	C17 H30 N10 O5 Cl
	489.2076	-1.8	-3.7	2.5	180.1	3.6	C16 H34 N6 O9 Cl
	489.2062	-0.4	-0.8	8.5	180.2	3.7	C13 H26 N16 O3 Cl
	489.2049	0.9	1.8	3.5	180.4	3.9	C12 H30 N12 O7 Cl
	489.2036	2.2	4.5	-1.5	180.6	4.1	C11 H34 N8 O11 Cl
	489.2022	3.6	7.4	4.5	180.8	4.3	C8 H26 N18 O5 Cl

Compound 11

Elemental Composition Report

Page 1

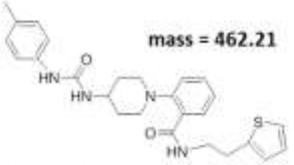

mass = 462.21

Single Mass Analysis

Tolerance = 10.0 PPM / DBE: min = -1.5, max = 50.0
Element prediction: Off
Number of isotope peaks used for i-FIT = 3

Monoisotopic Mass, Even Electron Ions
1337 formula(e) evaluated with 10 results within limits (all results (up to 1000) for each mass)
Elements Used:
C: 0-100 H: 0-120 N: 0-20 O: 0-20 S: 1-1
V030-7621 23 (0.942) Cm (21:23)
1: TOF MS ES+

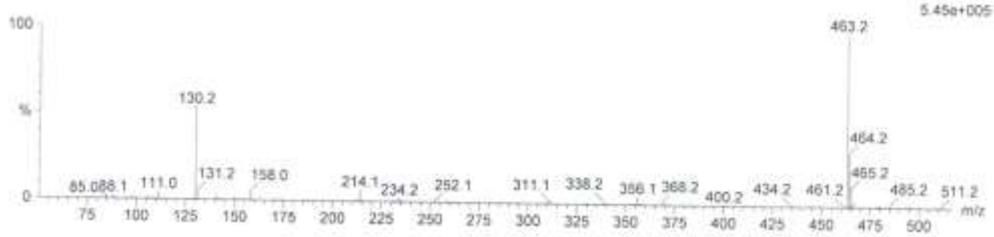

Minimum:				-1.5			
Maximum:		5.0	10.0	50.0			
Mass	Calc. Mass	mDa	PPM	DBE	i-FIT	i-FIT (Norm)	Formula
463.2162	463.2154	0.8	1.7	8.5	407.5	0.7	C35 H35 O6 S
	463.2168	-0.6	-1.3	13.5	407.6	0.8	C26 H31 N4 O2 S ✓MH+
	463.2208	-4.6	-9.9	17.5	410.2	3.4	C31 H31 N2 S
	463.2141	2.1	4.5	14.5	410.8	4.0	C22 H27 N10 S
	463.2128	3.4	7.3	9.5	411.7	4.9	C21 H31 N6 O4 S
	463.2200	-3.8	-8.2	5.5	414.6	7.8	C15 H31 N10 O5 S
	463.2186	-2.4	-5.2	0.5	415.3	8.5	C14 H35 N6 O9 S
	463.2173	-1.1	-2.4	6.5	416.3	9.5	C11 H27 N16 O3 S
	463.2159	0.3	0.6	1.5	416.7	9.9	C10 H31 N12 O7 S
	463.2133	2.9	6.3	2.5	418.0	11.2	C6 H27 N18 O5 S

Compound 12

Elemental Composition Report Page 1

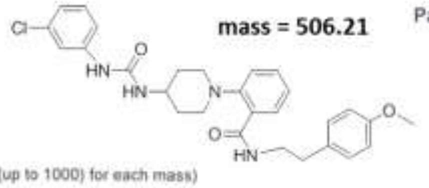

mass = 506.21

Single Mass Analysis
Tolerance = 10.0 PPM / DBE: min = -1.5, max = 50.0
Element prediction: Off
Number of isotope peaks used for i-FIT = 3

Monoisotopic Mass, Even Electron Ions
1531 formula(e) evaluated with 12 results within limits (all results (up to 1000) for each mass)
Elements Used:
C: 0-100 H: 0-120 N: 0-20 O: 0-20 Cl: 1-1
V030-7609 13 (0.536) Cm (10:14)
1: TOF MS ES+

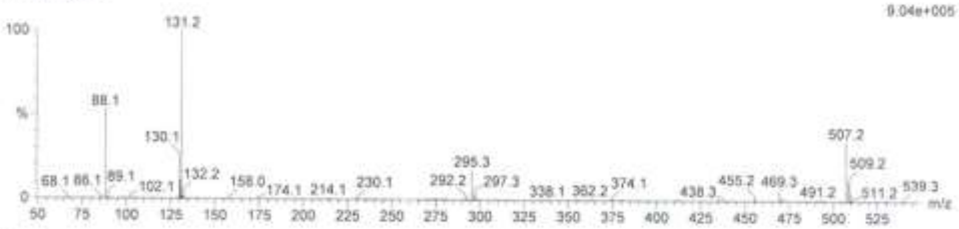

Mass	Calc. Mass	mDa	PPM	DBE	i-FIT	i-FIT (Norm)	Formula
Minimum:				-1.5	*		
Maximum:		5.0	10.0	50.0	*		
507.2152	507.2163	-1.1	-2.2	14.5	420.3	0.1	C28 H32 N4 O3 Cl ✓MH+
	507.2150	0.2	0.4	9.5	422.9	2.7	C27 H36 O7 Cl
	507.2136	1.6	3.2	15.5	424.0	3.8	C24 H28 N10 O Cl
	507.2123	2.9	5.7	10.5	427.7	7.6	C23 H32 N6 O5 Cl
	507.2195	-4.3	-8.5	6.5	428.8	8.6	C17 H32 N10 O6 Cl
	507.2109	4.3	8.5	5.5	430.1	9.9	C22 H36 N2 O9 Cl
	507.2181	-2.9	-5.7	1.5	430.2	10.0	C16 H36 N6 O10 Cl
	507.2181	-2.9	-5.7	12.5	430.2	10.0	C14 H24 N20 Cl
	507.2168	-1.6	-3.2	7.5	431.4	11.2	C13 H28 N16 O4 Cl
	507.2155	-0.3	-0.6	2.5	432.4	12.3	C12 H32 N12 O8 Cl
	507.2128	2.4	4.7	3.5	434.4	14.2	C8 H28 N18 O6 Cl
	507.2114	3.8	7.5	-1.5	435.2	15.0	C7 H32 N14 O10 Cl

Compound 13

Elemental Composition Report

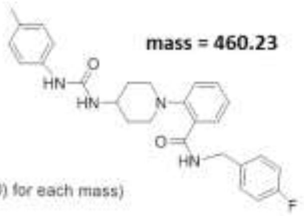

Page 1

Single Mass Analysis
Tolerance = 10.0 PPM / DBE: min = -1.5, max = 50.0
Element prediction: Off
Number of isotope peaks used for i-FIT = 3

Monoisotopic Mass, Even Electron Ions
1348 formula(e) evaluated with 12 results within limits (all results (up to 1000) for each mass)
Elements Used:
C: 0-100 H: 0-120 N: 0-20 O: 0-20 F: 1-1
V002-8746 8 (0.332) Cm (6:10)
1: TOF MS ES+

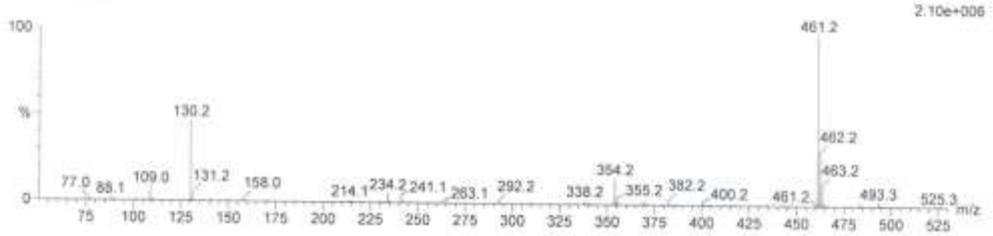

Minimum:				-1.5			
Maximum:		5.0	10.0	50.0			
Mass	Calc. Mass	mDa	PPM	DBE	i-FIT	i-FIT (Norm)	Formula
461.2357	461.2358	-0.1	-0.2	7.5	584.3	3.6	C12 H26 N16 O3 F
	461.2353	0.4	0.9	14.5	582.5	1.8	C27 H30 N4 O2 F ✓ [M+H]+
	461.2344	1.3	2.8	2.5	584.7	3.9	C11 H30 N12 O7 F
	461.2371	-1.4	-3.0	1.5	584.1	3.3	C15 H34 N6 O9 F
	461.2339	1.8	3.9	9.5	582.4	1.6	C26 H34 O6 F
	461.2385	-2.8	-6.1	6.5	583.8	3.1	C16 H30 N10 O5 F
	461.2326	3.1	6.7	15.5	583.0	2.3	C23 H26 N10 F
	461.2393	-3.6	-7.8	18.5	582.5	1.8	C32 H30 N2 F
	461.2318	3.9	8.5	3.5	585.3	4.6	C7 H26 N18 O5 F
	461.2398	-4.1	-8.9	11.5	583.6	2.9	C17 H26 N14 O F
	461.2398	-4.1	-8.9	0.5	583.4	2.7	C19 H38 O11 F
	461.2313	4.4	9.5	10.5	583.0	2.3	C22 H30 N6 O4 F

Compound 14

Elemental Composition Report

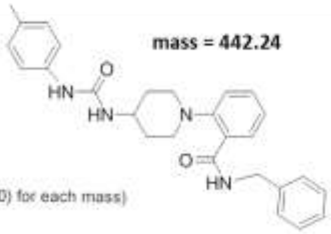

mass = 442.24

Page 1

Single Mass Analysis
Tolerance = 10.0 PPM / DBE: min = -1.5, max = 50.0
Element prediction: Off
Number of isotope peaks used for i-FIT = 3

Monoisotopic Mass, Even Electron Ions
1412 formula(e) evaluated with 10 results within limits (all results (up to 1000) for each mass)
Elements Used:
C: 0-100 H: 0-120 N: 0-20 O: 0-20
V010-6019 19 (0.775) Cm (16:19)
1: TOF MS ES+

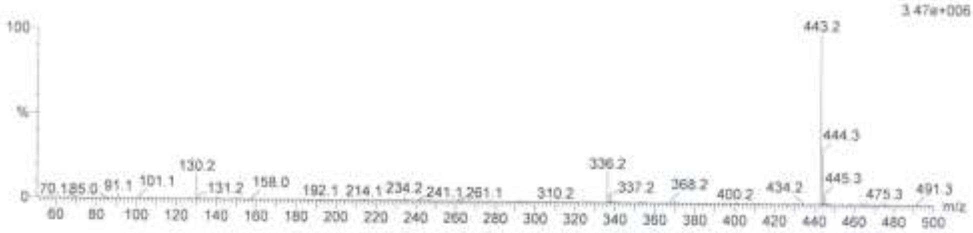

Minimum:				-1.5			
Maximum:		5.0	10.0	50.0			
Mass	Calc. Mass	mDa	PPM	DBE	i-FIT	i-FIT (Norm)	Formula
443.2442	443.2447	-0.5	-1.1	14.5	463.7	0.6	C27 H31 N4 O2 ✓ MH+
	443.2434	0.8	1.8	9.5	464.0	0.9	C26 H35 O6
	443.2420	2.2	5.0	15.5	467.9	4.8	C23 H27 N10
	443.2407	3.5	7.9	10.5	468.6	5.5	C22 H31 N6 O4
	443.2479	-3.7	-8.3	6.5	471.8	8.7	C16 H31 N10 O5
	443.2466	-2.4	-5.4	1.5	472.3	9.2	C15 H35 N6 O9
	443.2452	-1.0	-2.3	7.5	473.4	10.3	C12 H27 N16 O3
	443.2439	0.3	0.7	2.5	473.6	10.5	C11 H31 N12 O7
	443.2412	3.0	6.8	3.5	474.9	11.8	C7 H27 N18 O5
	443.2398	4.4	9.9	-1.5	475.1	12.0	C6 H31 N14 O9

Compound 15

Elemental Composition Report

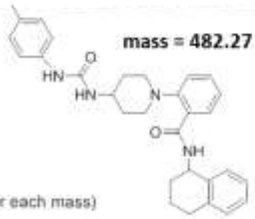

mass = 482.27

Page 1

Single Mass Analysis
Tolerance = 10.0 PPM / DBE: min = -1.5, max = 50.0
Element prediction: Off
Number of isotope peaks used for i-FIT = 3

Monoisotopic Mass, Even Electron Ions
1655 formula(e) evaluated with 13 results within limits (all results (up to 1000) for each mass)
Elements Used:
C: 0-100 H: 0-120 N: 0-20 O: 0-20
V030-7542 33 (1.348) Cm (33:38-9:20)
1: TOF MS ES+

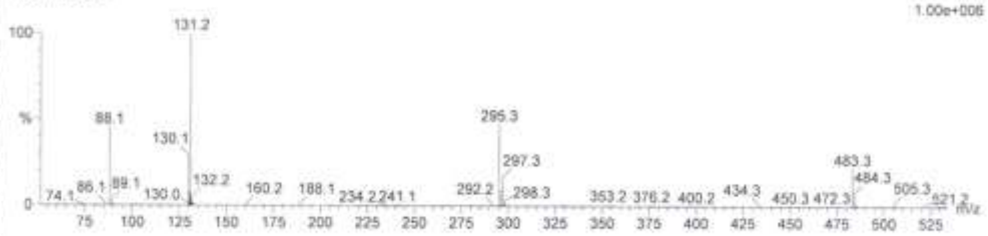

Mass	Calc. Mass	mDa	PPM	DBE	i-FIT	i-FIT (Norm)	Formula
Minimum:				-1.5			
Maximum:		5.0	10.0	50.0			
483.2768	483.2765	0.3	0.6	8.5	425.3	9.4	C15 H31 N16 O3
	483.2760	0.8	1.7	15.5	416.9	1.0	C30 H35 N4 O2
	483.2779	-1.1	-2.3	2.5	424.0	8.0	C18 H39 N6 O9
	483.2752	1.6	3.3	3.5	425.6	9.6	C14 H35 N12 O7
	483.2747	2.1	4.3	10.5	416.5	0.6	C29 H39 O6
	483.2792	-2.4	-5.0	7.5	423.4	7.5	C19 H35 N10 O5
	483.2738	3.0	6.2	-1.5	426.0	10.1	C13 H39 N8 O11
	483.2800	-3.2	-6.6	19.5	419.2	3.2	C35 H35 N2
	483.2733	3.5	7.2	16.5	420.3	4.4	C26 H31 N10
	483.2805	-3.7	-7.7	1.5	422.2	6.3	C22 H43 O11
	483.2805	-3.7	-7.7	12.5	423.1	7.2	C20 H31 N14 O
	483.2725	4.3	8.9	4.5	427.1	11.2	C10 H31 N18 O5
	480.2720	4.8	9.9	11.5	420.9	4.9	C25 H35 N6 O4

Compound 16

Elemental Composition Report Page 1

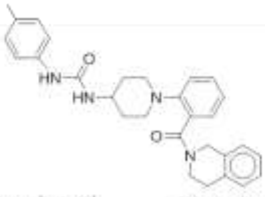

mass = 468.25

Single Mass Analysis
Tolerance = 10.0 PPM / DBE: min = -1.5, max = 50.0
Element prediction: Off
Number of isotope peaks used for i-FIT = 3

Monoisotopic Mass, Even Electron Ions
1568 formula(e) evaluated with 13 results within limits (all results (up to 1000) for each mass)
Elements Used:
C: 0-100 H: 0-120 N: 0-20 O: 0-20
V028-5777 55 (2.235) Cm (54:57-16:30)
1: TOF MS ES+

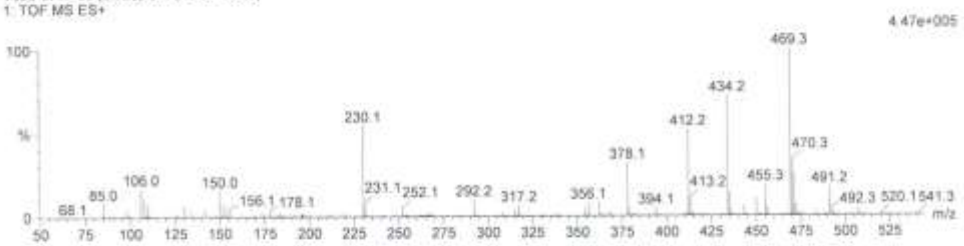

Minimum:				-1.5			
Maximum:		5.0	10.0	50.0			

Mass	Calc. Mass	mDa	PPM	DBE	i-FIT	i-FIT (Norm)	Formula
469.2607	469.2644	-3.7	-7.9	19.5	426.3	1.6	C34 H33 N2
	469.2590	1.7	3.6	10.5	426.7	1.9	C28 H37 O6
	469.2604	0.3	0.6	15.5	426.7	2.0	C29 H33 N4 O2 ✓ MH+
	469.2649	-4.2	-9.0	1.5	427.1	2.4	C21 H41 O11
	469.2563	4.4	9.4	11.5	427.2	2.5	C24 H33 N6 O4
	469.2577	3.0	6.4	16.5	427.2	2.5	C25 H29 N10
	469.2635	-2.8	-6.0	7.5	427.6	2.9	C18 H33 N10 O5
	469.2649	-4.2	-9.0	12.5	427.6	2.9	C19 H29 N14 O
	469.2622	-1.5	-3.2	2.5	427.7	3.0	C17 H37 N6 O9
	469.2595	1.2	2.6	3.5	428.3	3.6	C13 H33 N12 O7
	469.2609	-0.2	-0.4	8.5	428.3	3.6	C14 H29 N16 O3
	469.2582	2.5	5.3	-1.5	428.4	3.6	C12 H37 N8 O11
	469.2568	3.9	8.3	4.5	428.9	4.2	C9 H29 N18 O5

Compound 17

Elemental Composition Report

Page 1

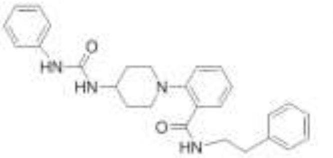

mass = 442.24

Single Mass Analysis

Tolerance = 10.0 PPM / DBE: min = -1.5, max = 50.0
Element prediction: Off
Number of isotope peaks used for i-FIT = 3

Monoisotopic Mass, Even Electron Ions
1412 formula(e) evaluated with 11 results within limits (all results (up to 1000) for each mass)
Elements Used:
C: 0-100 H: 0-120 N: 0-20 O: 0-20
VD10-1446 6 (0.259) Cm (2:8-36:50)
1: TOF MS ES+

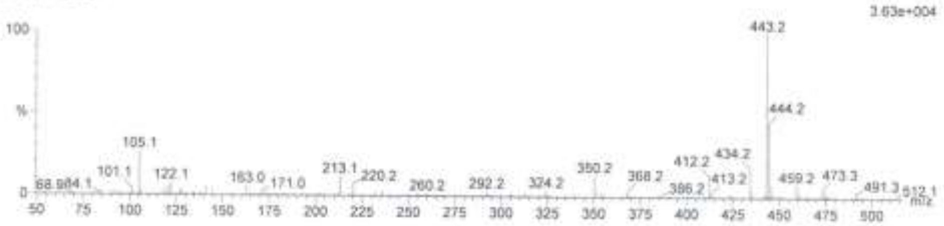

Mass	Calc. Mass	mDa	PPM	DBE	i-FIT	i-FIT (Norm)	Formula
Minimum:				-1.5			
Maximum:		5.0	10.0	50.0			
443.2435	443.2447	-1.2	-2.7	14.5	205.0	0.9	C27 H31 N4 O3 ✓ MH+
	443.2434	0.1	0.2	9.5	205.6	1.4	C26 H35 O6
	443.2420	1.5	3.4	15.5	206.1	2.0	C23 H27 N10
	443.2407	2.8	6.3	10.5	206.5	2.4	C22 H31 N6 O4
	443.2393	4.2	9.5	5.5	206.9	2.8	C21 H35 N2 O8
	443.2479	-4.4	-9.9	6.5	207.9	3.8	C16 H31 N10 O5
	443.2466	-3.1	-7.0	1.5	208.1	4.0	C15 H35 N6 O9
	443.2452	-1.7	-3.8	7.5	208.6	4.5	C12 H27 N16 O3
	443.2439	-0.4	-0.9	2.5	208.8	4.7	C11 H31 N12 O7
	443.2412	2.3	5.2	3.5	209.5	5.4	C7 H27 N18 O5
	443.2398	3.7	8.3	-1.5	209.6	5.5	C6 H31 N14 O9

Compound 18

Elemental Composition Report

Page 1

Single Mass Analysis
Tolerance = 10.0 PPM / DBE: min = -1.5, max = 50.0
Element prediction: Off
Number of isotope peaks used for i-FIT = 3

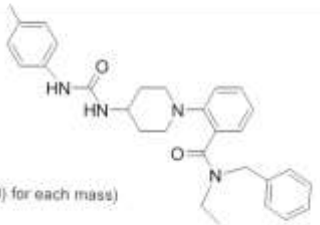

mass = 470.27

Monoisotopic Mass, Even Electron Ions
1584 formula(e) evaluated with 12 results within limits (all results (up to 1000) for each mass)
Elements Used:
C: 0-100 H: 0-120 N: 0-20 O: 0-20
V005-1856 44 (1.791) Cm (44:47-5:20)
1: TOF MS ES+

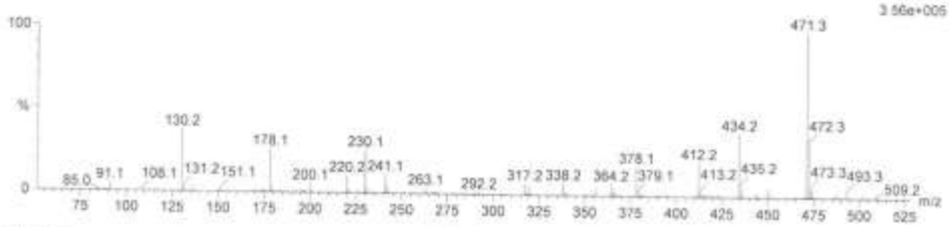

Minimum:				-1.5			
Maximum:		5.0	10.0	50.0			
Mass	Calc. Mass	mDa	PPM	DBE	i-FIT	i-FIT (Norm)	Formula
471.2762	471.2760	0.2	0.4	14.5	449.5	0.1	C29 H35 N4 O2 ✓ MH+
	471.2747	1.5	3.2	9.5	452.8	3.3	C28 H39 O6
	471.2800	-3.8	-8.1	18.5	453.8	4.3	C34 H35 N2
	471.2733	2.9	6.2	15.5	456.7	7.3	C25 H31 N10
	471.2805	-4.3	-9.1	0.5	457.7	8.2	C21 H43 O11
	471.2720	4.2	8.9	10.5	458.0	8.5	C24 H35 N6 O4
	471.2805	-4.3	-9.1	11.5	459.5	10.0	C19 H31 N14 O
	471.2792	-3.0	-6.4	6.5	459.8	10.4	C18 H35 N10 O5
	471.2779	-1.7	-3.6	1.5	460.4	11.0	C17 H39 N6 O9
	471.2765	-0.3	-0.6	7.5	462.4	12.9	C14 H31 N16 O3
	471.2752	1.0	2.1	2.5	462.7	13.3	C13 H35 N12 O7
	471.2725	3.7	7.9	3.5	464.7	15.2	C9 H31 N18 O5
