# Supplementary figures and images for: Discovery of (phenylureido)piperidinyl benzamides as prospective inhibitors of bacterial autolysin E from Staphylococcus aureus
Source: J Enzyme Inhib Med Chem. 2018 Aug 24;33(1):1239–47. doi: 10.1080/14756366.2018.1493474 (PMC6116672; doi:10.1080/14756366.2018.1493474)

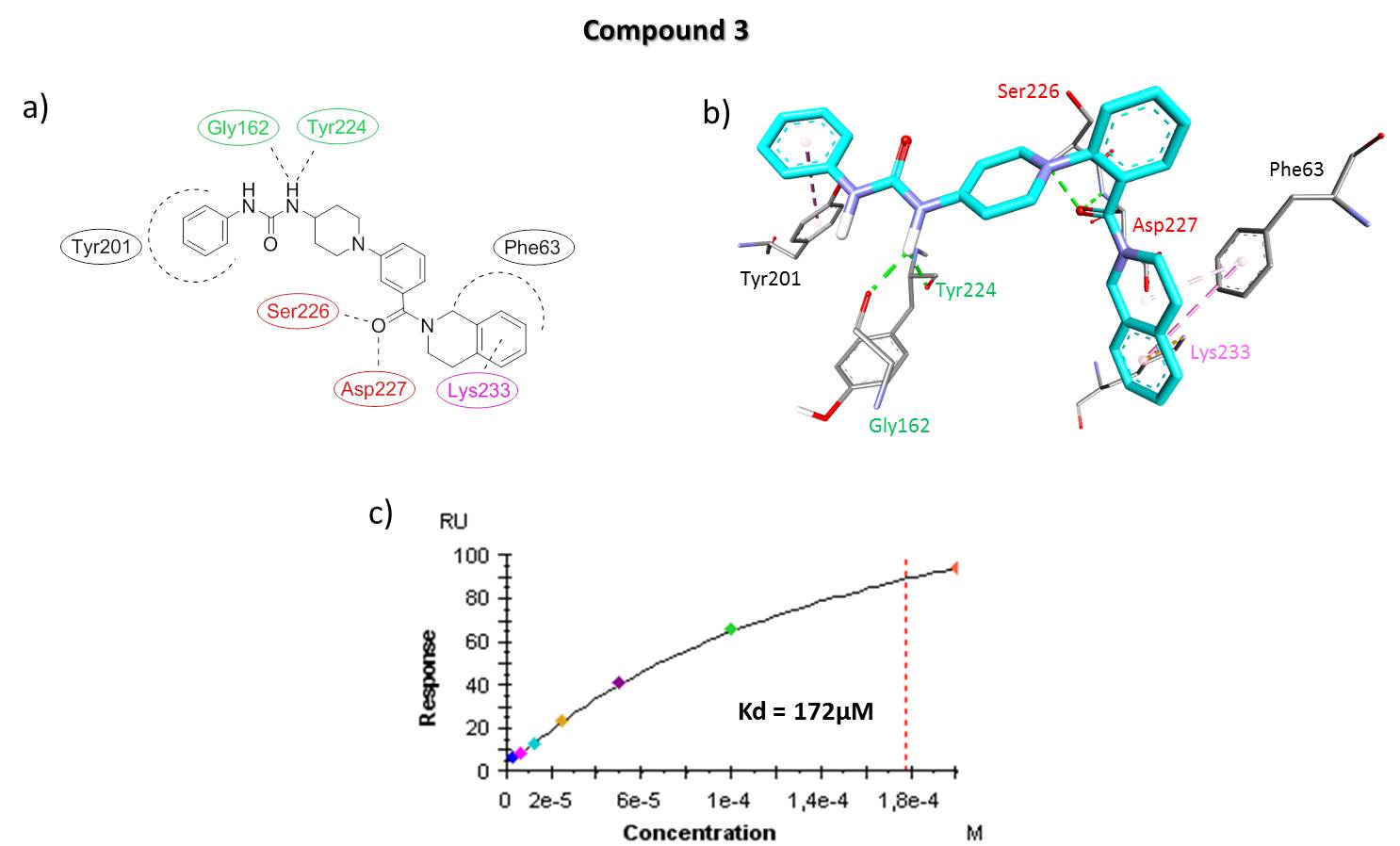

Supplement: Supplemental Material [file IENZ_A_1493474_SM1691.zip › IENZ_1493474_Supplementary Material/FigureS1.jpg]

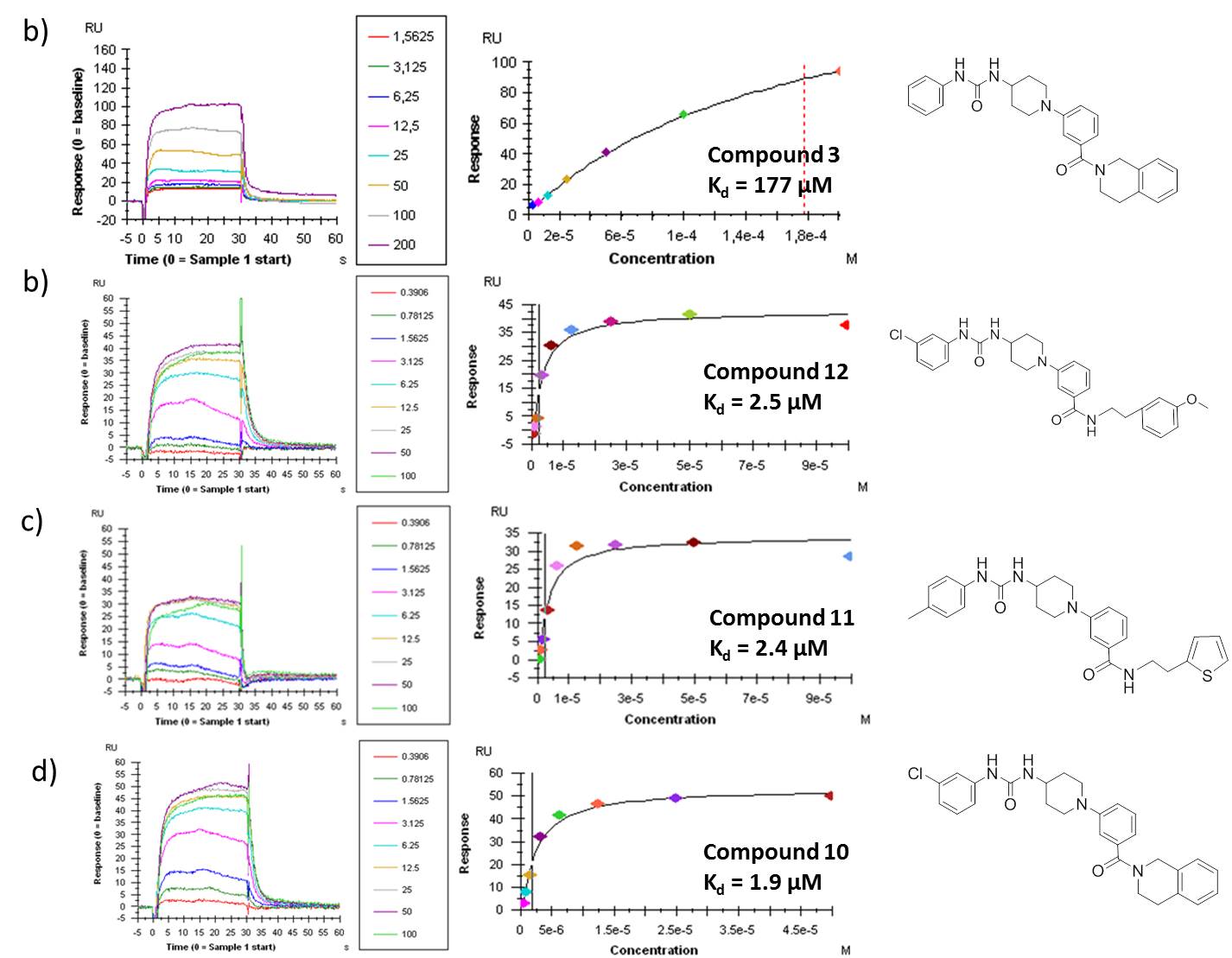

Supplement: Supplemental Material [file IENZ_A_1493474_SM1691.zip › IENZ_1493474_Supplementary Material/FigureS2.jpg]

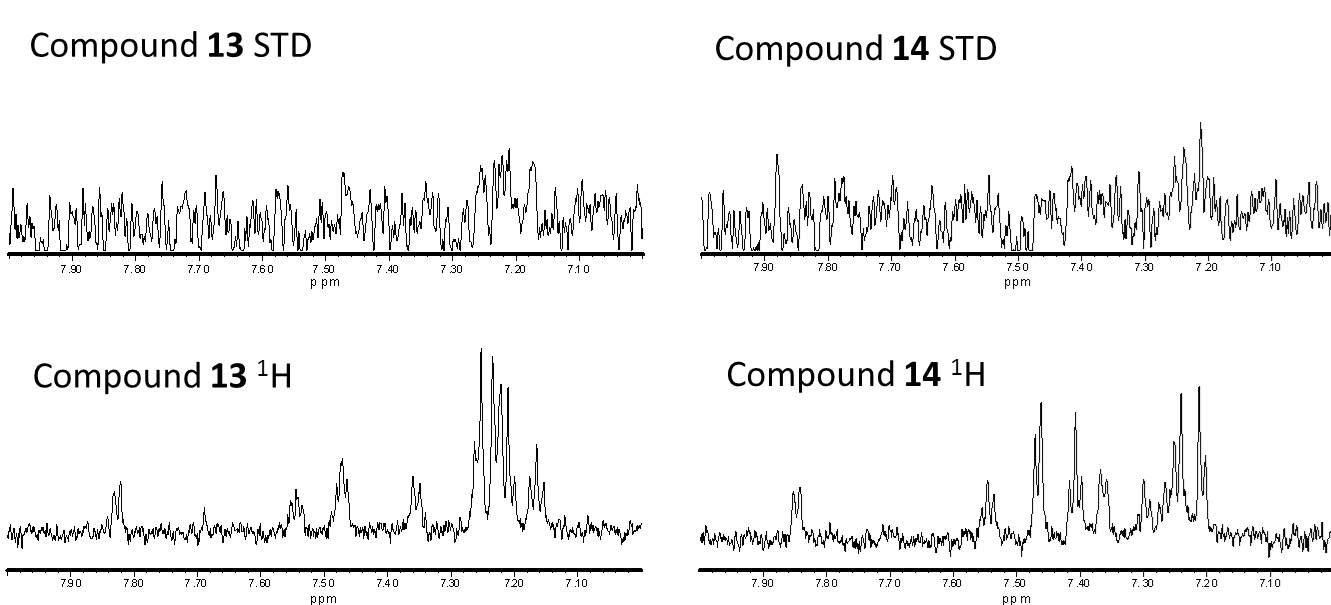

Supplement: Supplemental Material [file IENZ_A_1493474_SM1691.zip › IENZ_1493474_Supplementary Material/FigureS3.jpg]
